# Absorptive capacity facilitates adaptation to novel environmental disasters

**DOI:** 10.1371/journal.pone.0259368

**Published:** 2021-11-17

**Authors:** So-Min Cheong, Valentina A. Assenova

**Affiliations:** 1 Department of Geography and Atmospheric Science, University of Kansas, Lawrence, KS, United States of America; 2 Management Department, The Wharton School, University of Pennsylvania, Philadelphia, PA, United States of America; Universiti Pertahanan Nasional Malaysia, MALAYSIA

## Abstract

Absorptive capacity–the ability to learn and apply external knowledge and information to acquire material resources–is an essential but overlooked driver in community adaptation to new and unprecedented disasters. We analyzed data from a representative random sample of 603 individuals from 25 coastal communities in Louisiana affected by the Deepwater Horizon oil spill. We used simultaneous equation models to assess the relationship between absorptive capacity and resource acquisition for affected individuals after the disaster. Results show that the diversity of individuals’ prior knowledge coupled with the community’s external orientation and internal cohesion facilitate resource use. They go beyond simply providing resources and demonstrate individual and community features necessary for absorbing information and knowledge and help devise adaptation strategies to address the dynamics of changing economic, social, and political environment after the disaster.

## Introduction

An increase in environmental disasters worldwide compounded by an increase in the intensity and frequency of extreme climatic events leads to a higher probability of their occurrence in different locations [1–4]. Heatwaves, drought, fire, tornadoes, and floods ignore geographic boundaries and are increasingly occurring in areas previously immune to such disasters. In addition, complex technological systems generate a higher probability of “normal” accidents [5], such as oil spills. More places experience strange extreme events in greater magnitude or intensity, posing severe challenges for people. Adaptation to a diversity of environmental disasters is, therefore, an increasingly important global environmental issue [6]. To date, while there is an understanding that experience of a disaster can improve adaptation so that a repetition of the same type of disaster again has less impact, few studies have systematically examined how people and communities adapt to new or different disasters [7,8].

People’s previous experience of disasters shapes how they respond to future disasters. It can create vulnerabilities by way of path dependency that negatively affects the ability to respond to a disaster of a different type or magnitude. For example, a close-knit group’s self-belief and collective reinforcement of experience make it difficult for a community to prepare for heatwaves and drought that exceed their expectations [9,10]. People also tend to come back and rebuild in flood-prone zones partly because they are attached to their location. This “sense of place” is frequently engineered because of social capital [11,12] and hinders relocation from places of high risk [13]. Such bonding social capital can foster path dependence to the extent that community know-how and networks become self-referential [14,15]. In ordinary times, bonding social capital may work well and enhance solidarity. However, in times of crisis and change, it may result in a closed society lacking innovation and the diversity of resources, including knowledge necessary for adapting to change. If a cohesive community interacts mainly with its members, homophily abounds, preventing access to outside resources and entities [16,17]. Prevailing beliefs about the positive effects of cohesion can create barriers to resource absorption, particularly when affected communities require external assistance.

External assistance with resources after disasters are common. Environmental disasters often exceed the local capacity to cope and invite external engagement in response to disasters and extreme events [18–20]. Indicative of this is the inflow of external aid and expertise and the emergence of new groups for both short-term coping and longer-term adaptation. Complex impacts of environmental disasters such as oil spills and extreme climatic events require scientific expertise that communities often do not possess and call for the involvement of various government agencies to engage with communities [21,22]. While communities tend to self-organize immediately after the disaster before the external assistance arrives, subsequent issues of compensation and restoration are critical, for instance, after large oil spills and require sustained external resources inclusive of knowledge and information.

### Absorbing a new disaster

The necessity for external resources heightens unprecedented disasters that are different in type or magnitude than what a locality has experienced. The BP Deepwater Horizon oil spill was a new and unprecedented disaster for the affected people accustomed to hurricanes in Louisiana. It accompanied a different regulatory framework that activated the Oil Pollution Act in place of the Stafford Act, privately funded compensations instead of federal assistance, and large-scale federal environmental damage assessments and restoration projects. Coping with this novel disaster required behavioral change by all those affected, including states and communities, to accommodate the changed regulations and know-how related to the spill response and recovery [23]. It widened the gap between what people were used to and expected and what was required [24]. To bridge this gap, obtaining and using information and knowledge relevant to the largest spill in U.S. history was necessary for affected communities and their members to recover.

Adaptation to rapid environmental change employing external knowledge and information mirrors the findings in the literature on organizational innovation, which supports the importance of external resources including information and knowledge to promote adaptation and innovation in a dynamic and rapidly changing environment [25–27]. Cohen and Levinthal [27] state that the success of adaptation depends on a firm’s absorptive capacity, i.e., the capability to assimilate new information and to recognize and link new knowledge to existing in-house expertise. The concept of absorptive capacity at the community level complements discourse on the adaptive capacity of communities by advancing the concrete ways that communities and their members absorb external resources. As adaptive capacity literature has emphasized the necessity of raising the skills and knowledge of the local population to the new challenges of environmental change [28,29], assessing absorptive capacity has become even more critical. The focus on absorptive capacity also helps resolve knowledge transfer in disaster management as available knowledge is not used effectively or ignored [30–32].

**Fig 1:**
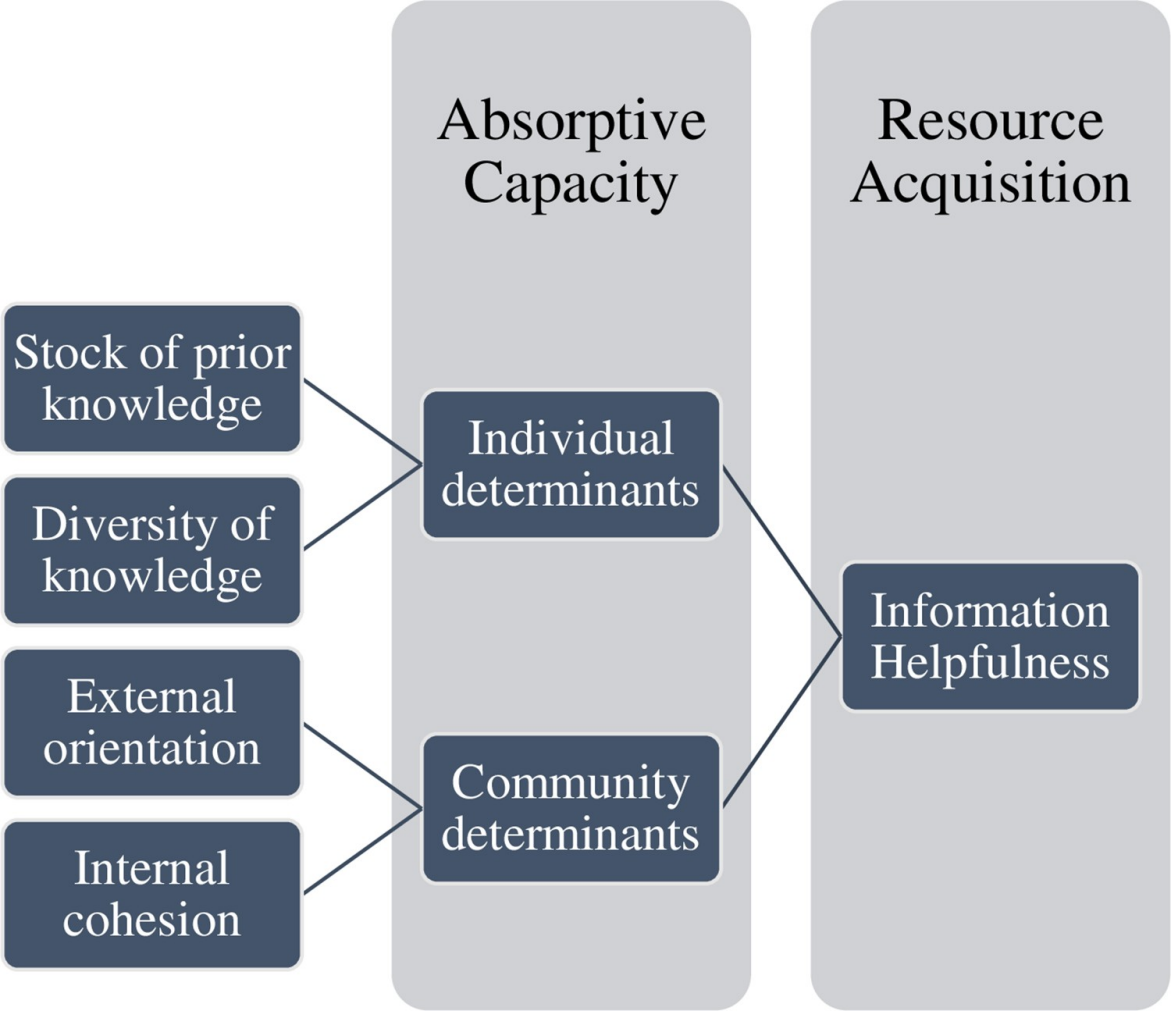
Individual and community determinants of absorptive capacity and resource acquisition after disasters. Conceptual model of the absorptive capacity construct. The model shows that the stock of prior knowledge, diversity of knowledge, external orientation, and internal cohesion explain individual variation in absorptive capacity (information helpfulness), a key determinant of resource acquisition after novel disasters (probability of filing claims).

The main question here is: Who put the new information to use and acquired resources? The key factors to observe are individuals’ prior related and diverse knowledge and the community’s internal cohesion and external orientation (Fig 1). The most critical component is the individual’s capacity to recognize the value of external knowledge utilizing one’s prior related and diverse knowledge. Prior knowledge derives from cumulative learning over time, diverse knowledge from exposure to different knowledge types and sources. This knowledge facilitates associations with uncertain and novel information that individuals and organizations sense from the environment. This process equates with sensing and seizing opportunities that are often latent [33]. To transfer and exploit knowledge after recognizing its value, the balance of inward and outward orientations of the organization is critical. The external orientation of the organization scans the environment for valuable information. In contrast, the internal orientation of the organization facilitates effective communication within the organization to share knowledge and know-how [34,35].

## Method

We apply absorptive capacity utilized in organizational theory to local communities affected by the Deepwater Horizon oil spill. Our analyses evaluated data from 32 item phone surveys of 603 adults from 25 coastal communities in Louisiana using representative sampling from August 11 to August 22, 2017. Measurements of absorptive capacity derived from Cohen and Levinthal’s pioneer work focus on characteristics of individuals and organizations that enhance capitalizing on new resources. The outcome in our survey, therefore, is resource acquisition. That is, if an individual needed help following the spill, did she or he get it. The receipt of helpful information predicts resource acquisition. The ability to capitalize on resources requires that we absorb information and then act on it (i.e., make it helpful). The predictors of getting this help are the stable characteristics of individuals and communities that predict the effectiveness of information use and, thereby, successful recovery.

### Data

SSRS, a survey and market research firm, completed data collection and data processing on behalf of the University of Kansas from August 11 through August 22, 2017. SSRS used random stratified sampling to survey by phone a representative sample of 603 adults in four coastal areas (25 zip-codes) of Louisiana highly affected by the DWH oil spill. Forty percent of sampled respondents from the survey responded to the landline frame and 60 percent to the cellular frame. In total, we obtained 242 phone surveys via landline and 361 via cell phone. The survey used Computer-Assisted Telephone Interviewing (CATI) software. The survey firm made up to five follow-up attempts to contact non-responsive numbers (e.g., no answer, busy, answering machine). Respondents could schedule a callback time at their convenience. SSRS provided anonymized data to the authors for data analysis. This project is approved by the University of Kansas Institutional for Review Board.

An individual’s selection into the study depended on his/her status as an affected party (and therefore a target beneficiary of recovery efforts). This status depended on whether an individual self-reported that s/he was affected by the BP oil spill (Q1 = “Yes”). The wording of the question posed was, “In 2010, there was an oil spill in the Gulf of Mexico, often referred to as the Deepwater Horizon oil spill. Were you affected by the spill?” The variable takes on a value of 1 if a surveyed respondent said he or she was affected and 0 otherwise. We use this variable to condition the estimates of the model by restricting estimates of outcomes to only those respondents who said that they were affected by the oil spill.

We compare the demographic characteristics for respondents comprising the sample population and U.S. Census data on the population distribution for age, gender, race, and education in the affected areas. The weighted sample population had the same distribution of demographic characteristics as the general population living within the affected areas in terms of age, gender, race and ethnicity, phone status, and education. Our sample had comparatively low education levels, with about 62 percent of respondents having achieved only a high-school level of education or less and fewer than 25 percent having some college education. Further, close to 75 percent of respondents self-identified as white (non-Hispanic). The largest groups of respondents by age and gender were women aged 45–64 (17.2 percent of the sample) and men aged 45–64 (17 percent of the sample).

### Measures

We operationalized individuals’ absorptive capacity as their acquisition, assimilation, and exploitation of financial/material and informational resources provided after the oil spill. The measures in our analyses were defined and measured as follows.

#### Absorptive capacity

We measured individuals’ absorptive capacity as a categorical variable indicating the extent of perceived helpfulness of the new information provided about dispersants, cleanup, and compensation claims after the spill. The wording of the question (Q12) in the survey was, “How helpful was the new information you learned in responding to the spill?” The categories followed a Likert scale ranging from 1 = “Not at all” to 5 = “A great deal,” with higher values indicating greater perceived helpfulness.

#### Resource acquisition

We measured affected individuals’ resource acquisition after the disaster as a binary variable indicating whether an individual reported that s/he received the necessary resources for recovery. The wording of the question (Q4) in our survey was, “During and after the spill, were you able to get the resources you needed to recover?” The variable takes on a value of 1 if a surveyed respondent said “Yes,” and 0 otherwise.

#### Individual prior knowledge of disaster response

We measured an individual’s stock of prior knowledge as a categorical variable indicating the extent of an individual’s prior knowledge about dispersants, cleanup, and compensation claims before the spill. The wording of the question (Q10) in our survey was, “Before the spill, how much did you know about dispersants, cleanup, and compensation claims?” The categories followed a Likert scale ranging from 1 = “Not at all” to 5 = “A great deal,” with higher values indicating greater knowledge.

#### Individual information source diversity

We measured individuals’ diversity of knowledge as a continuous variable indicating the frequency of interactions that an individual had with various sources of information, including NGOs and government agencies. The survey asked how frequently an individual interacted with each of the sources of information. The wording of the question (Q3) in the survey was, “And now thinking back to the time of the Deepwater Horizon oil spill in 2010, how frequently did you interact with (ITEM) after the spill to interpret new information and knowledge? Would you say it was:” 1 = “Not at all” to 5 = “A great deal,” with higher values indicating more frequent interaction.

#### Community external orientation

A continuous variable measuring a community’s total frequency of cooperation with other communities, external information scanning, and reported usefulness of external sources. This variable was computed as the sum of the following variables. *External information scanning (Q21)* was a categorical variable measuring how much community leaders recognized new knowledge and information from external sources and shared it within the community. The question asked: “In general, how much do you think your community leaders recognize the usefulness of new knowledge and information and share them with other members of the community?” The response categories ranged from 1 “not at all” to 5 “a great deal.” *Usefulness of external sources (Q22)* was a categorical variable measuring the usefulness of new knowledge from external sources. The question asked: “To what extent do you think your community uses external sources to get information?” The response categories ranged from 1 “not at all” to 5 “a great deal.” *Cooperation with other communities (Q16)* was a binary variable measuring whether the community makes new arrangements to work together with other communities. The question asked: “After the spill, did your community have new arrangements with other communities or organizations to work together?” The response categories were 1 “yes” and 2 “no.”

#### Community cohesion

A continuous variable measuring a community’s total frequency of community-wide meetings and reported feelings of kinship and social favors among community members. This variable was computed as the sum of the following variables. *Frequency of meetings (Q23)* was a categorical variable measuring the frequency of community-wide meetings. The wording of the question was: “How often does your community meet to discuss ways to improve and seek new opportunities?” The responses ranged from 1 “Not at all” to 5 “extremely often.” *Feelings of kinship (Q25*, *Q26)* was a categorical variable measuring the extent of cohesion of the community, in terms of feeling that the community was like an extended family (Q25). The wording of the question posed was, “To what extent do you think your community is like an extended family?” The categories followed a Likert scale ranging from 1 = “Not at all” to 5 = “A great deal,” with higher values indicating greater perceived cohesion. A categorical variable measuring the extent of personal identification with one’s community in terms of feeling insulted if the community is insulted (Q26). The wording of the question posed was, “When someone criticizes your community, how much do you feel like it is a personal insult?” The categories followed a Likert scale ranging from 1 = “Not at all” to 5 = “A great deal,” with higher values indicating greater personal identification. *Favors (Q27)* was a categorical variable measuring the frequency of favors among individuals in the community. The wording of the question was, “How often do you and people in your neighborhood do favors for each other? For example, watch each other’s children, help with shopping, or lend gardening or house tools” The responses ranged from 1 “Basically every day” to 6 “not at all.”

### Statistical models

We estimated several sets of simultaneous equation models to assess the relationships between absorptive capacity and resource acquisition for affected individuals. These specifications were two-stage least-squares (2SLS) models. The 2SLS models comprised two interdependent stages of estimation that provide a means to evaluate the role of absorptive capacity in resource acquisition. The first stage modeled the effects of individual and community variables on absorptive capacity. The second stage estimated the effects of absorptive capacity on resource acquisition. The 2SLS models took the following functional form:

$$\phi_{i}=\alpha_{1}+Z_{i}\theta +X_{i}\Gamma +{\varepsilon_{1}}_{i}$$ (1)

$$y_{i}=\alpha_{2}+\beta_{2SLS}{\hat{\phi }}_{i}+X_{i}\Gamma +{\varepsilon_{2}}_{i}$$ (2)

In these models, ${\hat{\phi }}_{it}$ is the predicted value of the mediating variables for respondent *i*. The first-stage equation modeled the relationship between absorptive capacity, *Z_i_*, and the mediating variables, captured by the coefficients θ. The term α_1_ is the model intercept, X is a vector of control variables with coefficients Γ; ${\varepsilon_{1}}_{i}$ is the vector of the model residuals. We controlled for tenure, age, race/ethnicity, gender, and information understanding. At the second stage of the estimation, *y_i_* captures variation in resource acquisition outcomes. The term α_2_ is the model intercept, and the term β_2SLS_ is the effect of the mediating variables. Finally, the term X is the vector of controls, and the variables ${\varepsilon_{2}}_{it}$ is the vector of the model residuals in the second-stage models. We estimated these models in R.

## Results and discussion

### Individual determinants of absorptive capacity

**Fig 2:**
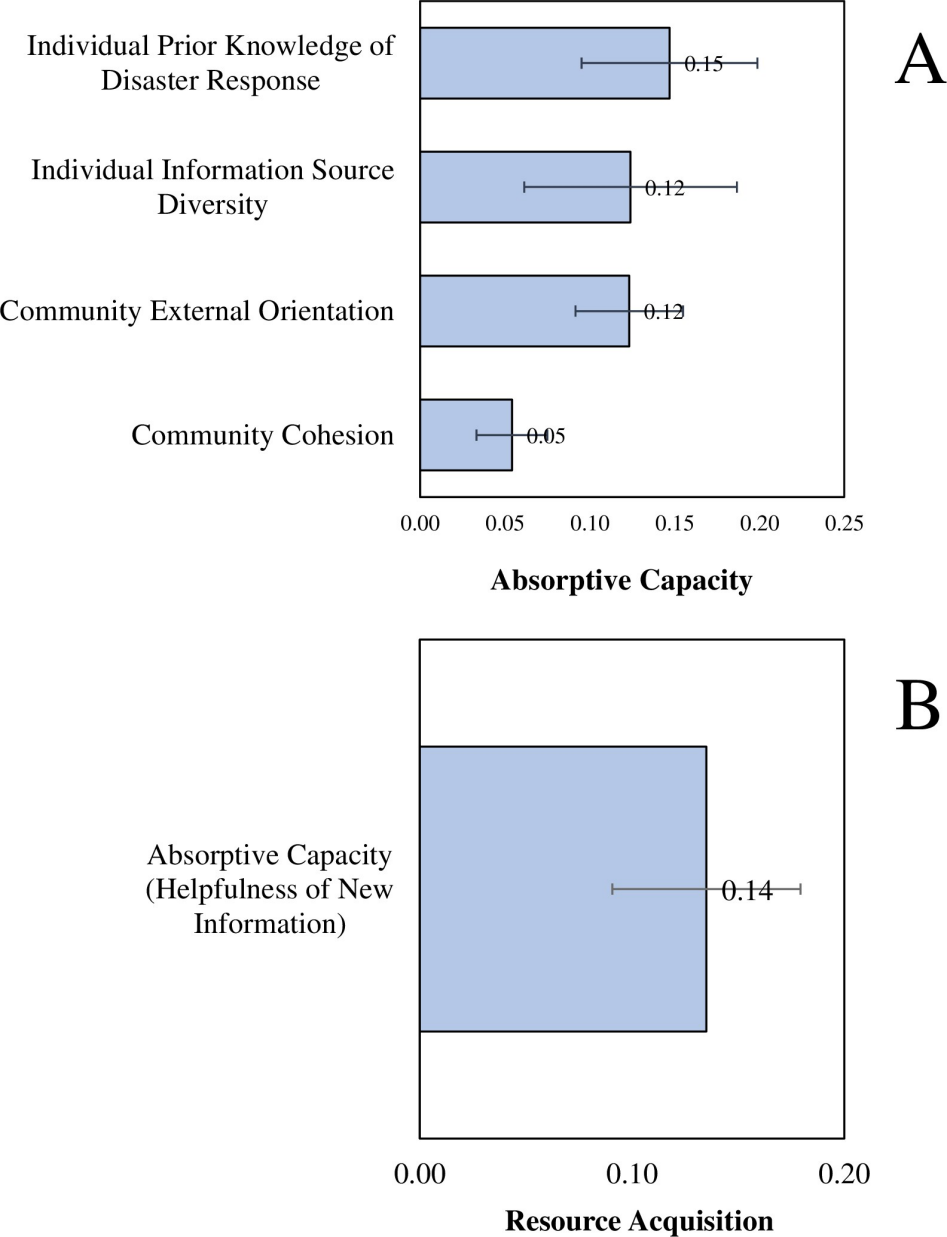
Determinants of absorptive capacity and resource acquisition after the Deepwater Horizon oil spill in Louisiana. Model coefficients and 95% CIs from structural equation models analyzing data from 603 survey responses of individuals in 25 coastal communities in Louisiana affected by the Deepwater Horizon Oil Spill. Panel (A) plots the results from the first-stage models, predicting absorptive capacity (helpfulness of new information). Panel (B) plots the results from the second-stage models, predicting resource acquisition (probability of filing claims).

Our results showed that absorptive capacity, measured as the helpfulness of new information related to the oil spill, depended on individual and community factors. We found that individuals’ prior knowledge of dispersants, cleanup, and compensation claims had a positive and statistically significant effect on how helpful they found the new information related to the oil spill to be (b = 0.15 [15%], se = 0.05, p = 0.005, Fig 2A). Every percent increase in their prior knowledge increased individuals’ perceived helpfulness of the new information provided by about 15 percent. This evidence is consistent with the idea that the greater the stock of prior knowledge, the more that individuals could learn and apply the new information in the wake of the oil spill, indicative of greater absorptive capacity.

Moreover, we also found that individuals’ reported helpfulness of new information increased with the number of different sources of information they had access to after the oil spill. Specifically, we found that the reported helpfulness of new information, indicative of absorptive capacity, increased by 12 percent with every one percent increase in the number of different sources for obtaining information after the oil spill (b = 0.12 [12%], se = 0.06, p = 0.05, Fig 2A). We interpret this result as evidence that the diversity of information networks helps to absorb new information. The diverse exposure to this new information leads to the greater likelihood that individuals tap into the diverse knowledge of the individual and help absorb the information at hand better. This evidence suggests that the value of new information provided in the wake of novel disasters increases with the number of different sources that disseminate this information, including local NGOs, government agencies, and other community organizations.

### Community determinants of absorptive capacity

We also found that individuals’ absorptive capacity depended on community factors, independent of the agencies and sources that provided the information. Two of these factors explained differences in how helpful individuals found the new information to be. First, a community’s external orientation, i.e., the ability to look to external sources of information after the disaster, explains greater absorptive capacity. Second, a community’s cohesion, i.e., the extent of connectedness and solidarity stemming from individuals’ sense of belonging to their community and relationships with other members of the community, predicts greater absorptive capacity. Both factors increased individuals’ ability to learn and apply novel knowledge and information.

A community’s external orientation included in our survey follows whether 1) communities used external sources to get information; 2) recognized the usefulness of new knowledge and information and shared it with other community members; and 3) cooperated with other communities after the spill. We found that individuals in communities with a higher level of external orientations found the new information provided after the oil spill to be more helpful for their recovery. Every one percent increase in a community’s external orientation resulted in a 12 percent increase in the likelihood that individuals in that community found the new information to be helpful (b = 0.12 [12%], se = 0.03, p = 0.0001, Fig 2A). We interpret this evidence as indicative of the vital role of the community’s external orientation in recovery efforts.

Second, a community’s sense of internal connectedness and solidarity (i.e., cohesion) also drove higher information absorption at the individual level. We found that individuals in more cohesive communities were more likely to find the new information helpful. These communities met more often to discuss ways to improve and seek new opportunities. They also fostered a great sense of belonging as they felt the community was like an extended family that favored each other. These communities were substantially more likely to enable information absorption after the oil spill and thereby recovery. We found that every one percent increase in a community’s sense of cohesiveness corresponded to a 5 percent increase in the reported helpfulness of new information among individuals affected by the oil spill (b = 0.05 [5%], se = 0.02, p = 0.01, Fig 2A). This evidence suggests that a community’s sense of connectedness and solidarity facilitated individuals’ ability to learn and apply new information in response to the novel disaster.

### Absorptive capacity as a determinant of resource acquisition

Finally, we examined the effects of information helpfulness, indicative of individuals’ ability to learn and apply new information (i.e., absorptive capacity) in their ability to acquire resources as part of the recovery efforts. We used filing compensation claims with BP as an indicator of resource acquisition. Much of the information provided after the disaster was related to dispersants, cleanup, and compensation claims. Therefore, we examined how individuals’ perceived helpfulness of this information, as explained by their prior knowledge, information source diversity, external orientation, and community cohesion, affected their likelihood of acting on this information by filing for compensation claims. Filing for claims is the first step toward absorbing resources provided to help communities and individuals recover in the aftermath of environmental disasters.

We found that all the factors noted above contributed to higher reported information helpfulness, indicative of absorptive capacity, which explained individual-level variation in the probability of filing compensation claims after the disaster. Specifically, we found that every one percent increase in individuals’ reported helpfulness of new information provided after the oil spill was associated with a 14 percent increase in the likelihood of filing compensation claims (b = 0.14 [14%], se = 0.04, p = 0.002, Fig 2B). Individuals were more likely to recover after the oil spill when they retained prior knowledge about dispersants, cleanup, and compensation claims, formed diverse knowledge from multiple sources of information on the spill and belonged to the community with higher external orientation internal cohesion.

## Conclusions

These findings have direct implications about the types of individuals and communities most resilient to novel disasters. They suggest that individuals with prior and diverse knowledge specific to the disaster recover better. The community’s balanced external orientation and internal cohesion are essential determinants of resilience in the face of novel disasters by affecting how individuals within the community learn and apply new information to aid recovery. Overall, this evidence shows that learning at both the individual and community levels is crucial for community adaptation to novel environmental disasters. As communities face new and unexpected challenges, the ability to absorb information using the prior and diverse knowledge base and the community’s balance of external and internal capabilities to use the information and knowledge is far better than simply providing resources to recover. Indeed, these characteristics at both the individual and community levels affect whether external resources, including information and financial compensation, will be absorbed within the affected communities to enable recovery and adaptation.

## Supporting information

S1 File (DOCX)
