# Supplementary material for: Absorptive capacity facilitates adaptation to novel environmental disasters
Source: PLoS One. 2021 Nov 17;16(11):e0259368. doi: 10.1371/journal.pone.0259368 (PMC8598048; doi:10.1371/journal.pone.0259368)
Supplement: S1 File — (DOCX) [file pone.0259368.s001.docx]

Supplementary Materials for

Absorptive capacity facilitates adaptation to novel environmental disasters

So-Min Cheong^1^*† and Valentina A. Assenova^2^

Correspondence to: somin@ku.edu

**This PDF file includes:**

Supplementary Text

Supplementary Text

***Selection into the study.***  An individual's selection into the study depended on his/her status as an affected party (and therefore a target beneficiary of recovery efforts). This status depended on whether an individual self-reported that s/he was affected by the BP oil spill (Q1 = “Yes”). The wording of the question posed was, “In 2010, there was an oil spill in the Gulf of Mexico, often referred to as the Deepwater Horizon oil spill. Were you affected by the spill?” The variable takes on a value of 1 if a surveyed respondent said he or she was affected and 0 otherwise. We use this variable to condition the estimates of the model by restricting estimates of outcomes to only those respondents who said that they were affected by the oil spill.

***Measures.*** We operationalized individuals' absorptive capacity as their information acquisition, assimilation, and exploitation, affecting resource acquisition for recovery after the oil spill. The measures in our analyses were defined and measured as follows:

**Individual determinants**

**1. Prior Related Knowledge**

KNOWLEDGE RELATED TO OIL SPILLS (Q10) – a categorical variable measuring the extent of an individual's prior knowledge about dispersants, cleanup, and compensation claims before the spill. The wording of the question posed was, “Before the spill, how much did you know about dispersants, cleanup, and compensation claims?” The categories followed a Likert scale ranging from 1= “Not at all” to 5= “A great deal,” with higher values indicating greater knowledge.

**2. Information Diversity/Network**

DIVERSITY OF INFORMATION SOURCES (Q2) - a binary variable denoting the source that a person turned to for information. The wording of the question posed was, “Now, thinking generally about any crisis that you experienced and needed help or advice, which of the following people or organizations did you turn to for information?” The variable takes on a value of 1 if a person turned to a specific source of information and 0 otherwise.

FREQUENCY OF INTERACTIONS (Q3) - a categorical variable measuring how frequently an individual interacted with the sources of information. The wording of the question posed was, “And now thinking back to the time of the Deepwater Horizon oil spill in 2010, how frequently did you interact with (ITEM from Q2) after the spill to interpret new information and knowledge? Would you say it was:” 1= “Not at all” to 5= “A great deal,” with higher values indicating more frequent interaction.

**Community determinants**

**1. Community External Orientation**

EXTERNAL INFORMATION SCANNING (Q21) – a categorical variable measuring how much community leaders recognized new knowledge and information from external sources and shared it. The question asked: “In general, how much do you think your community leaders recognize the usefulness of new knowledge and information and share them with other members of the community?” The response categories ranged from 1 “not at all” to 5 “a great deal.”

USEFULNESS OF EXTERNAL SOURCES (Q22) – a categorical variable measuring the usefulness of new knowledge from external sources. The question asked: “To what extent do you think your community uses external sources to get information?” The response categories ranged from 1 “not at all” to 5 “a great deal.”

COOPERATION WITH OTHER COMMUNITIES (Q16) - a binary variable measuring whether the community makes new arrangements to work together with other communities. The question asked: “After the spill, did your community have new arrangements with other communities or organizations to work together?” The response categories were 1 “yes” and 2 “no.”

**2. Community Cohesion**

FREQUENCY OF MEETINGS (Q23) – a categorical variable measuring the frequency of community-wide meetings. The wording of the question was: “How often does your community meet to discuss ways to improve and seek new opportunities?” The responses ranged from 1 “Not at all” to 5 “extremely often.”

FEELINGS OF KINSHIP (Q25, Q26) - a categorical variable measuring the extent of cohesion of the community, in terms of feeling that the community was like an extended family (Q25). The wording of the question posed was, “To what extent do you think your community is like an extended family?” The categories followed a Likert scale ranging from 1= “Not at all” to 5= “A great deal,” with higher values indicating greater perceived cohesion. A categorical variable measuring the extent of personal identification with one's community in terms of feeling insulted if the community is insulted (Q26). The wording of the question posed was, “When someone criticizes your community, how much do you feel like it is a personal insult?” The categories followed a Likert scale ranging from 1= “Not at all” to 5= “A great deal,” with higher values indicating greater personal identification.

FAVORS (Q27) – a categorical variable measuring the frequency of favors among individuals in the community. The wording of the question was, “How often do you and people in your neighborhood do favors for each other? For example, watch each other's children, help with shopping, or lend gardening or house tools” The responses ranged from 1 “Basically every day” to 6 “not at all.”

**Controls**

TENURE (Q28) – a categorical variable measuring the time lived in one's community in years. The wording of the question posed was, “How long have you lived in your community?” The categories followed a scale ranging from 1= “Less than two years” to 6= “20 years or more.”

AGE (S3) – a categorical variable measuring the age in years of the respondent. The wording of the question posed was, “What is your age?” The categories followed a scale ranging from 1= “18-24” to 7= “65+.”

GENDER (S1)– a binary variable measuring the gender of the respondent. The survey administrator recorded the gender of the respondent based on the voice without reading a question. The categories were 1= “MALE” and 2= “FEMALE.”

RACE/ETHNICITY (Q31)– a categorical variable measuring the self-identified race/ethnicity of the respondent. The wording of the question posed was, “Do you consider yourself white, black or African American, Asian, Native American, Pacific Islander, mixed-race, or some other race?” The categories were 1= “White” to 7= “Other.”

INFORMATION UNDERSTANDING (Q11) – a categorical variable measuring the extent of an individual's understanding of new information provided about dispersants, cleanup, and compensation claims after the spill. The wording of the question posed was, “As you received new information about the spill, how much were you able to understand it?” The categories followed a Likert scale ranging from 1= “Not at all” to 5= “A great deal,” with higher values indicating greater understanding.
